# Update on genistein and thyroid: an overall message of safety

**DOI:** 10.3389/fendo.2012.00094

**Published:** 2012-07-31

**Authors:** Herbert Marini, Francesca Polito, Elena B. Adamo, Alessandra Bitto, Francesco Squadrito, Salvatore Benvenga

**Affiliations:** ^1^ Section of Physiology and Human Nutrition, Department of Biochemical, Physiological and Nutritional Sciences, University of Messina, Messina, Italy; ^2^ Section of Pharmacology, Department of Clinical and Experimental Medicine and Pharmacology, University of Messina, Messina, Italy; ^3^ Section of Endocrinology, Department of Clinical and Experimental Medicine and Pharmacology, University of Messina, Messina, Italy; ^4^ Master on Childhood, Adolescent and Women’s Endocrine Health, University of Messina, Messina, Italy; ^5^ Interdepartmental Program of Molecular and Clinical Endocrinology, and Women’s Endocrine Health, University Hospital of Messina, Messina, Italy

**Keywords:** genistein, isoflavones, soy, safety, thyroid

## Abstract

Genistein aglycone, one of the soy isoflavones, has been reported to be beneficial in the treatment of menopausal vasomotor symptoms, osteoporosis, and cardiovascular diseases, as well as in a variety of cancers. However, issues of potential harm on thyroid function resulting from soy isoflavones consumption have been raised. Much of the evidence for the goitrogenic effects of isoflavones is derived from experimental *in vitro* and *in vivo* studies. Goitrogenic effects were also noted in infants fed non-iodine-fortified, soy-based formula, a problem that was easily solved with iodine fortification. Recent studies suggest that genistein shows a good profile of safety on the thyroid although definitive conclusions have not reached. The aim of this brief review is to summarize and better clarify the effects of genistein on human thyroid health.

Genistein (4′,5,7-trihydroxyflavone) is a phytoestrogen belonging to the class of soy isoflavones, a sub-class of flavonoids that have received great attention for their potential on human health benefits. Many epidemiological studies suggest that high dietary intake of soy is associated with lower incidence rates of certain forms of cancers (Setchell, 1998; Messina and Messina, 2010). Indeed, Asian women, whose diet is rich in isoflavones, have a low incidence of breast cancer as well as of other hormone-associated problems, such as osteoporosis and menopausal symptoms (Somekawa et al., 2001: Messina et al., 2004). Of the soy isoflavones, genistein ranks first in terms of mass of experimental and clinical studies performed. Genistein aglycone is an isoflavone found at low concentrations in soybeans but at high concentrations in certain soy-derived food. In contrast, genistin, the glucoside form of the aglycone genistein, is much more abundant in the unprocessed soybean. Structurally, genistein closely resembles 17β-estradiol (**Figure 1**), and indeed it binds to the estrogen receptors (ERs), the stronger affinity being for the ERβ isoform (Kuiper et al., 1998). Acting as a natural selective ER modulator, genistein exerts its estrogen agonist or antagonist action in a tissue- and dose-dependent manner (Setchell, 2001). Moreover, several *in vitro* and *in vivo* studies show that genistein aglycone has antineoplastic effects which stem from multiple actions: (a) modulation of cell growth and proliferation throughout tyrosine kinases and topoisomerase II inhibition, (b) stimulation of the immune system, (c) antiangiogenic effects, and (d) potent antioxidant capacity (Polkowski and Mazurek, 2000). Additionally, the anticancer property of genistein may be likely due to DNA methylation and/or chromatin modification (Li and Tollefsbol, 2010).

**FIGURE 1:**
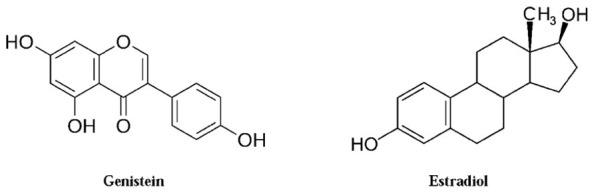
**Chemical structure of genistein and estradiol**.

Recently, clinical trials have been conducted to evaluate the benefit of genistein aglycone as a cure for menopausal vasomotor symptoms, osteoporosis, and cardiovascular disease. Specifically, administration of 54 mg/day genistein aglycone to postmenopausal women with low bone mass results in positive (beneficial) changes in vasomotor symptoms, bone mineral density and markers of bone turnover, and some predictors of cardiovascular risk without harmful estrogenic activity in the breast and uterus (Atteritano et al., 2007; Marini et al., 2007, 2008, 2010; D’Anna et al., 2009). In those studies, administration of pure genistein avoided possible interferences by other isoflavones and resulted in documented and stable increases of the isoflavone’s serum level. The promising safety profile of genistein aglycone may be a direct consequence of its greater affinity for the ERβ, which is particularly abundant in both the trabecular bone during the mineralization phase and the artery endothelial tissue, than ERα, which is more represented in the reproductive tissues (Nilsson and Gustafsson, 2011).

It is known that estrogens exert several effects on thyroid follicular cells, in that they modulate their cell cycle progression, proliferation, and function, thus potentially contributing to the pathogenesis of thyroid hyperplasia and even thyroid cancer. These effects are mediated by the binding to ERs as well as through distinct non-genomic molecular pathways (Ben-Rafael et al., 1987; Santin and Furlanetto, 2011). However, the expression of ERs in normal and neoplastic human thyroid tissues remains controversial probably because of inter-study differences in the methods used to quantify their expression. Another controversy of major concern is the different action of ERα and ERβ in neoplastic thyroid (Chen et al., 2008; Santin and Furlanetto, 2011).

Thyroid hormones regulate several metabolic processes and are crucial for normal growth, development, and maturation of the central nervous system, the cardiovascular system, and the skeleton in mammals. Decreased thyroid hormones synthesis and action results in impaired neuro-cognitive function and increased risk for cardiovascular diseases (Biondi and Cooper, 2008), thus impacting negatively on human health.

## EXPERIMENTAL STUDIES

Studies on the relationship between soybean intake and thyroid function have started about 80 years ago (Divi et al., 1997; Fitzpatrick, 2000; Doerge and Chang, 2002; Doerge and Sheehan, 2002); they suggest that consumption of soy and, specifically soy isoflavones as genistein, is goitrogenic and alters thyroid function.

The goitrogenic effects of genistein seem to derive from a direct interaction of this isoflavone with key pathways involved in thyroid hormones synthesis, metabolism, and thyroid hormone transport proteins (Radović, 2006). *In vitro* and *in vivo* studies showed that genistein is a potent inhibitor of thyroid peroxidase (TPO), a key enzyme in thyroid hormone synthesis (Divi and Doerge, 1996; Divi et al., 1997; Chang and Doerge, 2000; Doerge and Sheehan, 2002). Indeed, TPO catalyzes the iodination of thyroglobulin and oxidative coupling of diiodothyronine resulting in the thyroid hormone formation. Thus, inhibition of TPO leads to a reduction of thyroid hormones levels, with a subsequent increment of TSH release, that, in turn, provides a strong growth stimulus to the thyroid gland. Moreover, genistein also affects the metabolism of thyroid hormones and iodide re-utilization by inhibition of sulfotransferase enzymes (Ebmeier and Anderson, 2004).

Divi et al. (1997) demonstrated that the TPO-catalyzed iodination of tyrosine inhibited by genistein is dose-dependent; this effect reverses when sufficient amounts of iodide are added to incubation mixtures. Similar inhibition was observed in rat microsomal TPO and in rats feeding a diet fortified with different doses of genistein, but no changes were observed in blood sera levels of thyroid hormone or TSH (Chang and Doerge, 2000). Genistein is also known as a potent, dose-dependent, inhibitor of tyrosine kinase (Ravindranath et al., 2004), as well as thyroid hormone deiodination mediated by 5′-iodothyronine deiodinase (Mori et al., 1996; White et al., 2004).

Using preparations of liver enzymes, Ferreira et al. (2002) found that flavonoids other than genistein inhibited 5′-iodothyronine deiodinase. Indeed, *in vivo* experiments with the synthetic flavonoid EMD 21388 (Schröder-van der Elst et al, 1991), which inhibits thyroid hormone binding to plasma transthyretin, show a reduction of T3 content in tissues that express type II 5′-iodothyronine deiodinase.

More recently, Sosić-Jurjević et al. (2010) showed that a subcutaneous injection of 10 mg/kg of genistein or daidzein can disturb the pituitary-thyroid axis, causing hypothyroidism in orchidectomized middle-aged rats.

However, overall *in vitro* and *in vivo* data from animal studies are not easily to compare with data from human studies, as demonstrated by a recent paper (Setchell et al., 2011).

## HUMAN STUDIES

In humans, early studies showed that feeding infants with soy milk caused goiter in those with inadequate iodine intake even if this effect was reverted by iodine supplementation (Chorazy et al., 1995; Jabbar et al., 1997). Moreover, of 530 children aged 6–15 years living in iodine-deficient areas of India and consuming large amounts of flavonoids, almost all were goitrous (Brahmbhatt et al., 2000). This enormous rate of thyroid enlargement led the authors to conclude that goiter was due to the combined effect of iodine deficiency and flavonoid excess in their diet (Brahmbhatt et al., 2000).

A more recent human trial reports the effects of short-term soy consumption on thyroid parameters in relation with isoflavone levels in male and female healthy subjects (Hampl et al., 2008). After 7 days of soy consumption, levels of both genistein and daidzein were increased. The statistically significant relationships found at the end of soy consumption were: (i) between basal levels of daidzein and thyrotropin, (ii) between daidzein and antithyroglobulin in males, and (iii) between daidzein and free thyroxine in females. Genistein lacked any correlation with the above thyroid parameters. These results agree with a previous research in 268 children (Milerová et al., 2006). The authors investigated whether serum levels of genistein and daidzein were correlated with thyroid hormone function. This study showed only a modest association between isoflavones serum levels and parameters of thyroid function, such as free thyroxine, thyroglobulin antibodies, and thyroid volume.

It is well known that thyroid diseases are most common in women, especially during perimenopause and menopause, perhaps as consequence of an altered balance between estrogens and progesterone. Accordingly, the effects of genistein on thyroid function were also analyzed in postmenopausal women. Results from a 3-month study in postmenopausal women consuming an isoflavone-rich diet (containing 58% of total genistein) showed no significant effect of isoflavones on serum levels of thyroid hormones (Duncan et al., 1999).

Bruce et al. (2003) investigated thyroid function in 38 iodine-repleted postmenopausal women at baseline and after 90 and 180 days following supplementation with 90 mg (aglycone weight) of total isoflavones/day. Thyroid parameters did not differ between the placebo and the treatment arm.

In a 16-week duration study, 77 postmenopausal women were randomized to receive cow’s milk and a placebo supplement, soy milk and placebo supplement, or cow’s milk and isoflavone supplement (Ryan-Borchers et al., 2008). The results of this study are congruent with those of Bruce et al. (2003) and provide evidence that the levels of isoflavone intake do not adversely affect thyroid function, as indicated by serum TSH levels that remained within the normal range following the intervention period in all women; cognitive functioning in healthy postmenopausal women was also unaffected. A more recent clinical trial in postmenopausal women evaluated the effects of 3-year administration of pure genistein aglycone (54 mg/day) on thyroid-related markers (Bitto et al., 2010). Specifically, changes in thyroid hormone receptors expression, serum levels of thyroid hormones, and thyroid antibodies were assessed. The results showed that daily consumption of genistein aglycone did not modify circulating FT4, FT3, and TSH levels or thyroid antibodies. Furthermore, genistein aglycone administration over 3 years did not affect the expression of thyroid hormone receptors in peripheral blood mononuclear cells, thus confirming that genistein appears not to alter thyroid function in postmenopausal women.

A 12-week duration randomized, double-blind and placebo-controlled trial in 43 oophorectomized Indian women, evaluated the effect of 75 mg/day soy isoflavones (genistein and genistin 25%; daidzein and daidzin 15%) on serum levels FT3, FT4, TSH, TBG, and anti-TPO antibodies (Mittal et al., 2011). The only variation found was a modest decrease in serum FT3.

Finally, a recent study in men with localized prostate cancer addressed the safety of genistein in the male gender (Lazarevic et al., 2011). Genistein was administered at the dose of 30 mg/day for 3–6 weeks prior to prostatectomy, and thyroid hormones levels were measured as secondary outcome. Serum levels of thyroid hormones remained statistically unchanged.

## CONCLUSION

Overall, there is a scarcity of information about the effect of pure isoflavones, such as genistein, on thyroid safety in humans. Results of intervention trials are not easily comparable because the researchers have used (i) mixed isoflavones or isoflavone and protein mixtures with different dosage regimens, soy foods or supplements as the active treatment; (ii) the quality and amount of genistein varied widely in all of these previous studies; and (iii) the trials were of different duration. Although the overall evidence suggests that isoflavone genistein does not affect adversely thyroid function in euthyroid, iodine-replete individuals, further studies are warranted to better define the relationship between genistein and thyroid.

Infants and women deserve particular attention in order to assess the safety of genistein and/or other isoflavones on thyroid function, also considering that thyroid disorders are age- and gender-related.

## Conflict of Interest Statement

FS has received research support from Primus Pharmaceuticals for work on genistein. FS and AB are co-inventors on patents referring to genistein activity.

The remaining authors declare that the research was conducted in the absence of any commercial or financial relationships that could be construed as a potential conflict of interest.
