# Corrigendum: Update on genistein and thyroid: an overall message of safety

**DOI:** 10.3389/fendo.2022.1073400

**Published:** 2022-11-03

**Authors:** Herbert Marini, Francesca Polito, Elena B. Adamo, Alessandra Bitto, Francesco Squadrito, Salvatore Benvenga

**Affiliations:** ^1^ Section of Physiology and Human Nutrition, Department of Biochemical, Physiological and Nutritional Sciences, University of Messina, Messina, Italy; ^2^ Section of Pharmacology, Department of Clinical and Experimental Medicine and Pharmacology, University of Messina, Messina, Italy; ^3^ Section of Endocrinology, Department of Clinical and Experimental Medicine and Pharmacology, University of Messina, Messina, Italy; ^4^ Master on Childhood, Adolescent and Women’s Endocrine Health, University of Messina, Messina, Italy; ^5^ Interdepartmental Program of Molecular and Clinical Endocrinology, and Women’s Endocrine Health, University Hospital of Messina, Messina, Italy

**Keywords:** genistein, isoflavones, soy, safety, thyroid

In the published article, the Conflict of Interest statement was incorrectly filed. The correct Conflict of Interest statement appears below.

## Conflict of interest

FS has received research support from Primus Pharmaceuticals for work on genistein. Authors FS and AB are co-inventors on patents referring to genistein activity.

The remaining authors declare that the research was conducted in the absence of any commercial or financial relationships that could be construed as a potential conflict of interest.

The authors apologize for this error and state that this does not change the scientific conclusions of the article in any way. The original article has been updated.

## Publisher’s note

All claims expressed in this article are solely those of the authors and do not necessarily represent those of their affiliated organizations, or those of the publisher, the editors and the reviewers. Any product that may be evaluated in this article, or claim that may be made by its manufacturer, is not guaranteed or endorsed by the publisher.

